# Evaluation of the Antidiabetic Activity and Chemical Composition of *Geranium collinum* Root Extracts—Computational and Experimental Investigations

**DOI:** 10.3390/molecules22060983

**Published:** 2017-06-13

**Authors:** Sodik Numonov, Salamet Edirs, Khayrulla Bobakulov, Muhammad Nasimullah Qureshi, Khurshed Bozorov, Farukh Sharopov, William N. Setzer, Haiqing Zhao, Maidina Habasi, Mizhgona Sharofova, Haji Akber Aisa

**Affiliations:** 1 Key Laboratory of Plant Resources and Chemistry in Arid Regions, Xinjiang Technical Institute of Physics and Chemistry, Chinese Academy of Sciences, Urumqi 830011, China; sodikjon82@gmail.com (S.N.) salamet619@gmail.com(S.E.); khurshedbek@gmail.com (K.B.); shfarukh@mail.ru (F.S.); haiqing_zhq@126.com (H.Z.); maidn@ms.xjb.ac.cn (M.H.); 2 State Scientifically-Experimental and Production Organization, Academy of Sciences of the Republic of Tajikistan, Dushanbe 734063, Tajikistan; 3 University of Chinese Academy of Sciences, Beijing 100049, China; 4 Institute of the Chemistry of Plant Substances, Academy of Sciences of the Republic of Uzbekistan, Tashkent 100170, Uzbekistan; khayrulla@rambler.ru; 5 Department of Chemistry, Abdul Wali Khan University, Mardan 23200, Pakistan; mnasimuq@yahoo.com; 6 Department of Pharmaceutical Technology, Avicenna Tajik State Medical University, Rudaki Ave. 139, Dushanbe 734003, Tajikistan; 7 Department of Chemistry, University of Alabama in Huntsville, Huntsville, AL 35899, USA; setzerw@uah.edu; 8 Institute of Avicenna’s Medicine and Pharmacology, Academy of the Tajik Traditional Medicine, Dushanbe 734062, Tajikistan; mijgona72@mail.ru

**Keywords:** *Geranium collinum* Steph, polyphenolic compounds, NMR, PTP-1B, α-glucosidase, molecular docking

## Abstract

The root of *Geranium collinum* Steph is known in Tajik traditional medicine for its hepatoprotective, antioxidant, and anti-inflammatory therapeutic effects. The present study was conducted to evaluate of potential antidiabetic, antioxidant activities, total polyphenolic and flavonoid content from the different extracts (aqueous, aqueous-ethanolic) and individual compounds isolated of the root parts of *G. collinum*. The 50% aqueous-ethanolic extract possesses potent antidiabetic activity, with IC_50_ values of 0.10 μg/mL and 0.09 μg/mL for the enzymes protein-tyrosine phosphatase (1B PTP-1B) and α-glucosidase, respectively. Phytochemical investigations of the 50% aqueous-ethanolic extract of *G. collinum*, led to the isolation of ten pure compounds identified as 3,3′,4,4′-tetra-*O*-methylellagic acid (**1**), 3,3′-di-*O*-methylellagic acid (**2**), quercetin (**3**), caffeic acid (**4**), (+)-catechin (**5**), (–)-epicatechin (**6**), (–)-epigallocatechin (**7**), gallic acid (**8**), β-sitosterol-3-*O*-β-d-glucopyranoside (**9**), and corilagin (**10**). Their structures were determined based on 1D and 2D NMR and mass spectrometric analyses. Three isolated compounds exhibited strong inhibitory activity against PTP-1B, with IC_50_ values below 0.9 μg/mL, more effective than the positive control (1.46 μg/mL). Molecular docking analysis suggests polyphenolic compounds such as corilagin, catechin and caffeic acid inhibit PTP-1B and β-sitosterol-3-*O*-β-d-gluco-pyranoside inhibits α-glucosidase. The experimental results suggest that the biological activity of *G. collinum* is related to its polyphenol contents. The results are also in agreement with computational investigations. Furthermore, the potent antidiabetic activity of the 50% aqueous-ethanolic extract from *G. collinum* shows promise for its future application in medicine. To the best of our knowledge, we hereby report, for the first time, the antidiabetic activity of *G. collinum.*

## 1. Introduction

In recent years herbal medicines have started to gain importance as a source of hypoglycemic agents. It is estimated that more than one thousand plant species are being used as folk medicine for diabetes [1]. The biological effects of the plants or herbal products used as alternative medicines to treat diabetes are related to their chemical composition. Polyphenols are not only capable of reducing oxidative stress, but also inhibiting carbohydrate-hydrolyzing enzymes, thus preventing hyperglycemia [2,3]. Herbal products rich in flavonoids, coumarins, terpenoids and other constituents help to reduce blood glucose levels [4].

Protein tyrosine phosphatase (PTP-1B), is a negative regulator of insulin signaling; it inhibits insulin-stimulated tyrosine phosphorylation of insulin receptor (IR), insulin receptor substrate-1 (IRS-1) and is a therapeutic target for type 2 diabetes [5]. Acarbose, as an inhibitor of α-glucosidase, has been shown to be non-toxic and to be a weak antidiabetic agent. The inhibitory effects of naturally occurring substances from plant materials on α-glucosidase have been well recognized, therefore, evaluation of bioactive constituents of plant origin represents a great challenge for the discovery of new diabetes drugs [6].

*Geranium collinum* Stephan ex Willd. is a perennial plant that belongs to the family Geraniaceae. In tropical and subtropical areas of the globe, there are 810 species in the family, which belong to eight genera. In Tajikistan there are more than 20 species, which belong to three genera, namely *Pelargonium* L’Hér., *Geranium* L. and *Erodium* L’Hér. *G. collinum* is one of the most common plants in Central Asia [7]. In Tajikistan, it grows in the wild areas of Zarafshan, Varzob and Hissar valley, Darwaz, Western Pamir and other regions of the country. *G. collinum* grows at an altitude of 800–2700 m above sea level, in the belts of black wood, juniper, mixed grass steppes and subalpine meadows, mainly along rivers and streams. The literature shows that the plant contains phenols (pyrogallol, catechol), saponins, tannins, carbohydrates (monosaccharides, sucrose, glucose, fructose, sorbose, rhamnose, arabinose, xylose, ribose, maltose, starch, hemicellulose), vitamin C and flavonoids (avicularin, quercetin, guayaverin, kaempferol) [8].

*G. collinum* has been used for the treatment of rheumatism, gout, dysentery, external and internal bleeding, as well as in the treatment of skin wounds, eczema, scabies, tenosynovitis and pruritus [9]. This plant has also been proven effect for curing pneumonia, catarrhal symptoms of the stomach and intestines, as well as in certain gynecological diseases [9]. Studies have been conducted to evaluate its hepatoprotective, antioxidant, anti-inflammatory and hypocholesterolemic action therapeutic effects in relation to its contents of total polyphenols and flavonoids [9]. The present work was performed to investigate the chemical constituents, molecular docking analysis, and antidiabetic potential of several root extracts, isolation of individual phenolic compounds and steroid glucosides of *G. collinum* by studying their effects on inhibition of α-glycosidase enzymes and protein-tyrosine phosphatase (PTP-1B). Additionally, antioxidant activity along with total polyphenolic compounds and total flavonoids contents in the root extracts of the *G. collinum* plant were also determined.

## 2. Results and Discussion

### 2.1. Optimization of the Extraction Procedure

**Figure 1:**
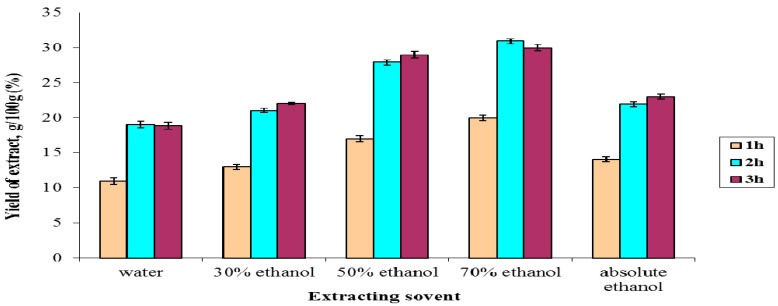
Graphical comparison of the yields of different extractions of the roots of *G. collinum*.

The method of extracting plant material under reflux was selected due to its routine application, requiring easily available apparatus and glassware, and high extractive yield. Different combinations of ethanol with water (30%, 50%, 70%, 100%) and pure water were evaluated in order to determine the corresponding extraction efficiencies and to identify the optimal solvent and time of extraction. Extraction was conducted for various extraction time durations using the optimal extraction conditions. The extract recovery yield appeared to increase as the extraction time increased up to 3 h, but further extension of the extraction time did not result in any further increase of extract recovery yield. No big difference in the extractive yield was observed between 2 h and 3 h and in the case of 70% ethanol 2 h extraction delivered more yield than 3 h. Therefore, in order to save energy and time, the optimal extraction time was set to be 2 h. All of the extracts obtained with 2 h extraction times were used for quantification of total polyphenols, flavonoids and their pharmacological activities. Figure 1 shows a graphical comparison of the extraction yields of various combinations of solvents and extraction time. Using 2 h extraction 70% ethanol delivered the highest extraction yield (31 g/100 g) among all the tested solvents, as the extraction values for H_2_O, 30%, 50%, and 100% ethanol were 19 g/100 g, 21 g/100 g, 28 g/100 g and 22 g/100 g, respectively.

### 2.2. Spectral Identification of the Isolated Compounds

**Figure 2:**
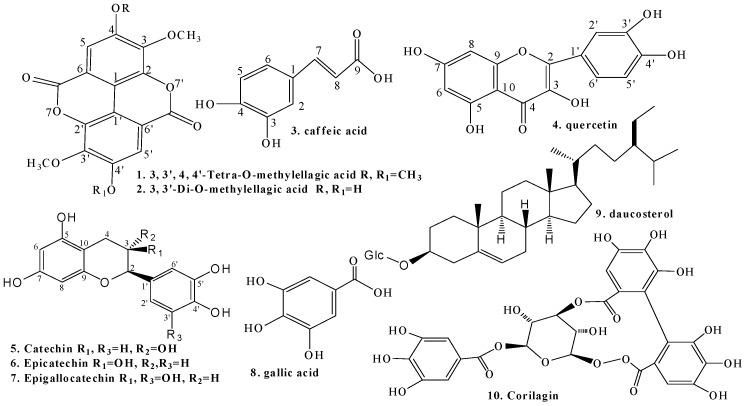
Chemical structures of the major compounds isolated from 50% ethanol extract of *Geranium collinum*.

The structures of all isolated compounds were elucidated by analyzing their spectral data (MS, ^1^H- and ^13^C-NMR, including HMQC, HMBC and DEPT) and by comparison with published literature data. They were thus identified as 3,3′,4,4′-tetra-*O*-methylellagic acid [10], 3,3′-di-*O*-methylellagic acid [11], caffeic acid [12], quercetin [13], (+)-catechin [14], (–)-epicatechin [13], (–)-epigallocatechin [15], gallic acid [16], β-sitosterol-3-*O*-β-d-glucopyranoside [17] and corilagin [18] (Figure 2).

### 2.3. Total Polyphenolic Compounds

Total polyphenolic compounds were estimated as gallic acid equivalents, using the Folin-Ciocalteu method, whereby polyphenolic compounds produce a blue-colored complex with a wavelength maximum at 740 nm. Seven point calibration curves were constructed using Microsoft Excel spreadsheets and forcing the curve through zero. The curve delivered a R^2^ value of 0.9956 and a straight line equation (*y* = 10.909*x*). The highest amounts of total polyphenolic compounds were calculated as 349.84 ± 0.21 and 180.14 ± 0.11 mg GAE/g of the dried extract in 50% ethanol and 70% ethanol extracts, respectively (Table 1). Total polyphenolic compounds quantified in water, 30% and absolute ethanol extracts were: 12.21 ± 0.10, 83.74 ± 0.18 and 100.42 ± 0.14 mg GAE/g of the dried extract, respectively.

### 2.4. Total Flavonoids Contents

The quantification of contents of total flavonoids was based on the complex formation between AlCl_3_ and flavonoids present in the extracts. Seven point calibration curve was obtained with an R^2^ value as 0.9971 producing a straight line equation (*y* = 9.157*x*). Using this equation, quantity of total flavonoids (x) with high contents in 50% and 70% ethanol extracts were determined as 96.07 ± 0.08 and 75.31 ± 0.07 mg quercetin equivalents (QE)/g of the dried extract (Table 1). In water, 30% and absolute ethanol extracts, the estimated total flavonoids contents were: 3.31 ± 0.04, 42.77 ± 0.12 and 55.68 ± 0.02 mg quercetin equivalents (QE)/g of the dried extract, respectively.

### 2.5. Antioxidant Activity

The antioxidant activity of several phenolic compounds such as catechin, gallic acid, caffeic acid and chlorogenic acid have been previously evaluated [19]. The majority of the antioxidant activities (such as free radical scavenging) of herbal medicines, vegetables and fruits may be due to their phenolic compounds. A comparative evaluation of the antioxidant activities of the extracts after 2 h of extraction is tabulated in Table 1. A lower IC_50_ value than the standard vitamin C implied a significant antioxidant activity of the tested samples. Among the understudied extracts the 30% ethanol extract was comparably active, with an IC_50_ value of 10.89 ± 0.63 μg/mL. The IC_50_ values (μg/mL) of the 50% ethanol, 70% ethanol, absolute ethanol and water extracts were: 11.21 ± 0.49, 12.69 ± 0.6, 11.23 ± 0.7 and 15.17 ± 0.84, respectively. 

### 2.6. Antidiabetic Activities of Crude Extracts and Isolated Individual Compounds

Flavonoids and phenolic glycosides of plant extracts have shown promising effect as inhibitors of PTP-1B and α-glucosidase. They can be used in pharmaceutical formulations against type 2 diabetes mellitus [14,20,21]. The antidiabetic effect of gallic acid was evaluated in vitro on inhibitory enzymes (α-glucosidase and α-amylase) [22]. Antidiabetic activity was determined using PTP-1B inhibition and α-glucosidase procedures. PTP-1B is a negative regulator of the insulin signaling pathway [23]. All extracts of *G. collinum* were obtained after a period of extraction of 2 h. They were assayed for their inhibitory activities against PTP-1B and α-glycosidase, delivering strong activity, with IC_50_ values ranging from 0.1 µg/mL to 1.98 µg/mL (Table 1). Among the five tested samples, water and 50% ethanol extracts showed strongest antidiabetic (PTP-1B inhibition) activity with an IC_50_ 0.13 ± 0.01 μg/mL and 0.10 ± 0.01 μg/mL, respectively. That is more effective than the positive control for the PTP-1B inhibitor (1.46 ± 0.40 μg/mL). Results of α-glucosidase inhibitory activities of root extracts are presented on Table 1. In addition, in vitro inhibitory effect on the enzymes PTP-1B and α-glucosidase of the pure isolated compounds **1**–**10** from *G. collinum* were evaluated. Catechin, epicatechin and corilagin delivered the strongest activities, with IC_50_ values of 0.62, 0.23 and 0.87, respectively. That was more than the known PTP-1B inhibitor (1.46 μg/mL). Catechin, epicatechin, corilagin and quercetin have shown potent antidiabetic activities in the α-glucosidase inhibition assay. IC_50_ values of the all the samples are presented in Table 2. The results of the in vitro assays, antidiabetic analyses of the isolated pure compounds, confirmed the results of the in silico computational investigation. It is well known that polyphenols can provide health benefits to humans attributed to their strong radical-scavenging and antioxidant effects [24]. These effects may also contribute to the prevention of diseases, such as diabetes. Additionally, intake of catechin-rich non toxic plants slightly inhibited postprandial elevation of blood glucose levels and oxidative products. Therefore these effect suggest that consumption of decoctions containing catechins could reduce the risk of type 2 diabetes.

The experimental results suggest promise for the future application of plant extracts for the development of phytopharmaceuticals and food formulations against diabetes. Nevertheless, toxicological studies should be carried out to ascertain the boundary between health beneficial effects and the risk of possible toxicity.

### 2.7. Molecular Docking

In order to provide some insight as to which compound(s) may be responsible for the antidiabetic activity, a molecular docking study was carried out on two different protein structures of human PTP-1B and two different protein structures of human α-glucosidase. Molegro re-rank energies (E_dock_) and normalized docking scores (DS_norm_) of the *G. collinum* ligands with the protein targets are summarized in Table 3.

Polyphenolic ligands such as epigallocatechin or caffeic acid showed preferential docking with HsPTP1B, although the docking energies were not as exothermic as the co-crystallized ligands for these proteins. Daucosterol (β-sitosterol-3-*O*-β-d-glucopyranoside), on the other hand, showed docking preference for α-glucosidase.

**Figure 3:**
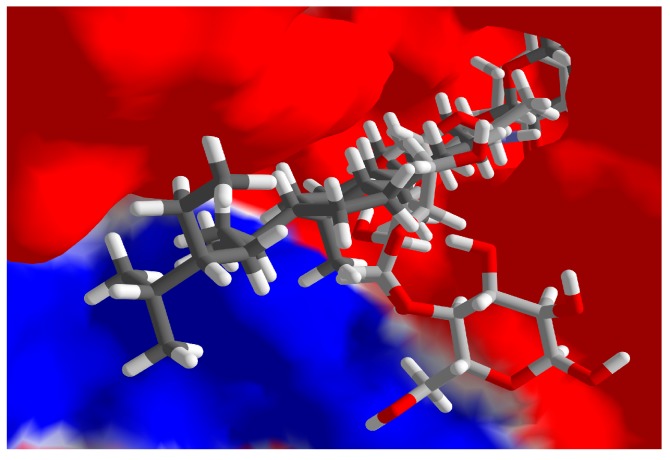
Lowest-energy docked pose of daucosterol (dark grey stick figure) with human maltase-glucoamylase (PDB 3TOP), showing the electrostatic surface of the binding site. The co-crystallized ligand, α-acarbose, is shown as a light grey stick figure.

The docking energy for daucosterol was comparable to the co-crystallized ligand, α-acarbose. The β-d-glucose moiety of daucosterol overlays the 6-amino-4-(hydroxymethyl)-4-cyclo-hexene-1,2,3-triol group of the co-crystallized α-acarbose, occupying a cavity on the protein surrounded by hydrogen-bonding amino acids His1584, Asp1526, and Asp1279 (Figure 3). Although corilagin showed strong docking to both PTP-1B and α-glucosidase, this compound is a hydrolyzable tannin, which is well known to bind indiscriminately to proteins and shows non-selective docking.

## 3. Experimental Section

### 3.1. General Procedures

Absolute ethanol and acetone were purchased from Tianjinshi Baishi Chemicals Company (Urumqi, China). DMSO, DPPH, pNPP, NaOH, NaCl, vitamin C, quercetin (98%), gallic acid (≥97%), aluminum chloride, sodium acetate and Folin-Ciocalteau reagent (2 N) were purchased from Sigma-Aldrich GmbH (Steinheim, Germany). All the chemicals and reagents were of analytical grade and double-distilled water was used throughout the experiment. NMR spectra recorded on Varian MR-400 (400 MHz for ^1^H and 100 MHz for ^13^C) spectrometer. Melting points were determined using a BUCHI Melting Point B-540 apparatus (Sigma-Aldrich, Darmstadt, Germany). Column chromatographic separation was performed on Sephadex LH-20 gel (Amersham Pharmacia Biotech, Stockholm, Sweden) and silica gel (100–200 mesh, Qingdao Haiyang Chemical Factory, Qingdao, China). The spots on the TLC plate were identified by spraying with 5% H_2_SO_4_ solution in EtOH and heating the plate at about 105 °C. UPLC analysis was performed on a Waters Acquity UPLC™ system (Waters, Milford, MA, USA) equipped with binary solvent delivery pump, an auto sampler, and a photodiode array detector (PAD). The instrument was controlled by the Waters Empower 2 software. The chromatographic separation was performed using a Waters Acquity BEH Shield C18 column (100 mm 2.1 mm i.d., 1.8 µm, Waters), operated at 35 °C. 

### 3.2. Plant Material

Underground parts of *G. collinum* were collected from Takob Valley in the Republic of Tajikistan in October 2015. The roots were authenticated by Professor Yusuf Nuraliev and voucher sample has been deposited in the herbarium of the Xinjiang Technical Institute of Physics and Chemistry Urumqi, Chinese Academy of Science.

### 3.3. Extraction and Isolation Procedures

Extraction efficiency was determined based on the total percent value of the amount extracted using different mixtures of aqueous ethanol (30%, 50%, 70%, 100%) and pure water employing the reflux extraction procedure. Each time root part of *G. collinum* (100 g) was weighed accurately and extracted with each of the aforesaid solvents (500 mL) for 1, 2 or 3 h. The extracts were reduced to dryness under reduced pressure. The dried residue of 50% aqueous ethanol extract (8 g) was mixed thoroughly with 32 g of dry silica gel (200–300 mesh). The mixture was dried in the oven at 45 °C and adsorbed on a 320 g silica gel column (1500 cm × 3.5 cm). Elution was performed in a gradient mode starting with petroleum ether−ethyl acetate (7:1). The polarity of the eluent was gradually increased using petroleum ether–ethyl acetate (4:1–0:1) and ethyl acetate−methanol (9:1–2:1), respectively. The fractions were analyzed by silica gel TLC using the mobiles phases (CHCl_3_/MeOH/H_2_O, 65:35:5 and 73:24:4). Spots were visualized by heating at 110 °C silica gel plates sprayed with 5% H_2_SO_4_ in EtOH. Similar fractions were combined after developing their thin layer chromatography (TLC) profiles, giving six fractions. Fraction 2 was applied onto a silica gel (100–200 mesh) column (750 cm × 3 cm) and eluted with chloroform–methanol (8:1) to yield compounds **1**, **2**, **3**. Compound **4** was obtained from combined fraction 3 on a Sephadex LH-20 (1200 cm × 1.5 cm) column by eluting with chloroform–methanol (4:1). Fraction 4 was purified with methanol–chloroform (9:1) on a Sephadex LH-20 (1200 cm × 1.5 cm) column to give compounds **5** and **6**. Pure compounds **7** and **8** were purified from fraction 5 with methanol–acetone (1:1) using Sephadex LH-20 (1200 cm × 1.5 cm) column chromatography. Compound **9** was isolated from fraction 6 using methanol–water (4:1) as eluent via Sephadex LH-20 (1200 cm × 1.5 cm) column chromatography. Compound **10** was isolated from fraction 5 via UPLC with PDA detector at the R_t_ = 18.26 min. The mobile phase consisted of 0.2% formic acid-water (A) and acetonitrile (B) with a gradient elution of 95% A (0–3 min), 95–90% A (3–20 min), 90–89% A (20–20.5 min), 89% A (20.5–29 min). Chromatograms were recorded at 254 nm and a flow rate of 250 µL·min^−1^ was used injecting 500 µL of the sample.

### 3.4. Characterization Data of Isolated Pure Compounds

*3,3′,4,4′-Tetra-O-methylellagic acid* (**1**). Colorless needle-like crystals (from pyridine), m.p. 223–225 °C, ^1^H-NMR (400 MHz, DMSO-*d*_6_): δ 7.65 (s, 2H, H-5,5′), 4.10 (s, 6H, OCH_3_), 4.12 (s, 6H, OCH_3_); ^13^C-NMR (100 MHz, DMSO-*d*_6_): δ 111.12 (C-1,1′), 140.92 (C-2,2′), 140.15 (C-3,3′), 153.74 (C-4,4′), 107.41 (C-5,5′), 113.29 (C-6,6′), 158.23 (C-7,7′), 60.97 (2 × OCH_3_), 56.69 (2 × OCH_3_).

*3,3*′*-Di-O-methylellagic acid* (**2**). Yellow powder (MeOH), m.p. 210–212 °C, ^1^H-NMR (400 MHz, DMSO-*d*_6_): δ 7.52 (s, 2H, H-5,5′), 4.04 (s, 6H, OCH_3_), 10.73 (br. s., 2H, OH); ^13^C-NMR (100 MHz, DMSO-*d*_6_): δ 111.59 (C-1,1′), 141.34 (C-2,2′), 140.36 (C-3,3′), 152.32 (C-4,4′), 111.59 (C-5,5′), 112.25 (C-6,6′), 158.60 (C-7,7′), 61.09 (2 × OCH_3_).

*Caffeic acid* (**3**). Pale yellow crystals (MeOH), m.p. 223–225 °C, ^1^H-NMR (400 MHz, DMSO-*d_6_*): δ_H_ 7.36 (1H, d, *J* = 15.9 Hz, H-7), 6.97 (1H, d, *J* = 2.0 Hz, H-2), 6.91 (1H, dd, *J* = 8.2; 2.0 Hz, H-6), 6.70 (1H, d, *J* = 8.2 Hz, H-5), 6.12 (1H, d, *J* = 15.9 Hz, H-8), ^13^C-NMR (100 MHz, DMSO-*d*_6_): δ_c_ 167.95 (C-9), 148.18 (C-4), 145.57 (C-3), 144.63 (C-7), 125.70 (C-1), 121.19 (C-6), 115.75 (C-5), 115.12 (C-8), 114.65 (C-2).

*Quercetin* (**4**). Yellow needles (MeOH), m.p. 314–315 °C, ^1^H-NMR (400 MHz, DMSO-*d*_6_): δ 6.17 (d, 1H, *J* = 2.2 Hz, H-6), 6.40 (d, 1H, *J* = 2.2 Hz, H-8), 6.86 (d, 1H, *J* = 8.5 Hz, H-5′), 7.51 (dd, 1H, *J* = 8.5; 2.2, Hz, H-6′), 7.65 (d, 1H, *J* = 2.2 Hz, H-2′), 12.47 (s, 1H, OH-5), 10.77 (br. s, 1H, OH-7), 9.59 (br. s, 1H, OH-3′), 9.36 (br. s, 1H, OH-3′), 9.30 (br. s, 1H, OH-4′); ^13^C-NMR (100 MHz, DMSO-*d*_6_): δ 146.79 (C-2), 135.72 (C-3), 175.84 (C-4), 160.72 (C-5), 98.18 (C-6), 163.89 (C-7), 93.36 (C-8), 156.13 (C-9), 103.01 (C-10), 121.95 (C-1′), 115.61 (C-2′), 145.06 (C-3′), 147.71 (C-4′), 115.05 (C-5′), 119.97 (C-6′). 

*(+)-Catechin* (**5**). Colorless crystals (MeOH), m.p. 228–229 °C, ^1^H-NMR (400 MHz, acetone-*d_6_*): δ 4.57 (d, 1H, *J* = 7.8 Hz, H-2), 4.00 (m, 1H, H-3), 2.91 (dd, 1H, *J* = 16.1; 5.5 Hz, H-4a), 2.53 (dd, 1H, *J* = 16.1; 8.4 Hz, H-4b), 6.03 (d, 1H, *J* = 2.2 Hz, H-6), 5.88 (d, 1H, *J* = 2.2 Hz, H-8), 6.90 (d, 1H, *J* = 1.9 Hz, H-2′), 6.80 (d, 1H, *J* = 8.1 Hz, H-5′), 6.76 (dd, 1H, *J* = 8.1, 1.9 Hz, H-6′), 3.90 (d, 1H, *J* = 4.8, OH-3), 8.17 (s, 1H, OH-5), 7.98 (s, 1H, OH-7), 7.82 (br.s, 1H, OH-3′), 7.88 (br. s, 1H, OH-4′); ^13^C-NMR (100 MHz, acetone-*d*_6_): δ 82.72 (C-2), 68.34 (C-3), 28.82 (C-4), 157.20 (C-5), 96.13 (C-6), 157.73 (C-7), 95.46 (C-8), 156.92 (C-9), 100.66 (C-10), 132.22 (C-1′), 115.24 (C-2′), 145.62 (C-3′), 145.69 (C-4′), 115.71 (C-5′), 120.07 (C-6′). 

*(–)-Epicatechin* (**6**). Pale red powder (MeOH), m.p. 224–226 °C, ^1^H-NMR (400 MHz, pyridine-*d*_5_): δ 5.40 (br. s., 1H, H-2), 4.75 (br. s., 1H, H-3), 3.44 (dd, 1H, *J* = 16.4; 4.6 Hz, H-4a), 3.57 (dd, 1H, *J* = 16.4; 3.7 Hz, H-4b), 6.71 (d, 1H, *J* = 2.3 Hz, H-6), 6.69 (d, 1H, *J* = 2.3 Hz, H-8), 7.30 (d, 1H, *J* = 8.1 Hz, H-5′), 7.37 (dd, 1H, *J* = 8.1; 2.0 Hz, H-6′), 7.95 (d, 1H, *J* = 2.0 Hz, H-2′), 11.26 (br. s., 4H, OH), 11.47 (br. s., 1H, OH); ^13^C-NMR (100 MHz, pyridine-*d*_5_): δ 80.59 (C-2), 67.43 (C-3), 30.18 (C-4), 159.20 (C-5), 97.14 (C-6), 159.12 (C-7), 96.28 (C-8), 158.12 (C-9), 100.68 (C-10), 132.64 (C-1′), 116.85 (C-2′), 147.43 (C-3′), 147.33 (C-4′), 116.60 (C-5′), 119.89 (C-6′).

*(–)-Epigallocatechin (3,3*′*,4,5,5*′*,7-flavanhexol)* (**7**). Colorless crystals (MeOH), m.p. 236–238 °C, ^1^H-NMR (400 MHz, acetone-*d_6_*): δ 4.52 (d, 1H, *J* = 7.4 Hz, H-2), 3.98 (1H, m, H-3), 2.87 (dd, 1H, *J* = 16.1; 5.4 Hz, H-4a), 2.53 (dd, 1H, *J* = 16.1; 8.2 Hz, H-4b), 6.02 (d, 1H, *J* = 2.3 Hz, H-6), 5.88 (d, 1H, *J* = 2.3 Hz, H-8), 6.46 (s, 2H, H-2′, H-6′), 3.92 (br. s, 1H, OH-3), 8.35–7.50 (m, 5H, OH); ^13^C-NMR (100 MHz, acetone-*d*_6_): δ 82.74 (C-2), 68.30 (C-3), 28.43 (C-4), 157.19 (C-5), 96.08 (C-6), 157.68 (C-7), 95.42 (C-8), 156.85 (C-9), 100.57 (C-10), 131.53 (C-1′), 107.19 (C-2′), 146.25 (C-3′), 133.29 (C-4′), 146.25 (C-5′), 107.19 (C-6′).

*Gallic acid* (**8**). Colorless needles (MeOH), m.p. 245–246 °C, ^1^H-NMR (400 MHz, CD_3_OD): δ 7.07 (2H, s, H-2, H-6); ^13^C-NMR (100 MHz, CD_3_OD): δ 170.38 (COOH), 146.3(C-3, C-5), 110.30 (C-2, C-6), 139.53 (C-4), 121.88 (C-1). 

*β-Sitosterol-3-O-β-d-glucopyranoside* (**9**). Colorless powder (MeOH/H_2_O), m.p. 210–211 °C. Compound **9** was identified by comparison with an authentic standard of β-sitosterol-3-*O*-β-d-glucopyranoside by TLC and comparision with published literature data.

*Corilagin (1-O-galloyl-3,6-O-hexahydroxydiphenoyl-β-d-glucose)* (**10**). Pale yellow powder (MeOH). The ^1^H- and ^13^C-NMR spectra of **10** showed characteristic signals for a hydrolyzable tannin with galloyl, 1-*O*-galloyl-3,6-(*R*)-hexahydroxydiphenoyl (HHDP) and glucose units. ^1^H-NMR (400 MHz, DMSO-*d*_6_): 7.03 (s, H-2 and H-6 galloyl proton), 6.59 (s, H-2′ HHDP unit), 6.50 (s, H-2′′ HHDP unit), 6.25 (d, *J* = 7.2 Hz, H-1′′′ glucose unit), 4.62 (brs, H-3′′′ glucose unit), 4.36 (t, *J* = 8.1 H, H-5′′′ glucose unit), 4.29–4.22 (m, H-6a′′′ and H-4′′′ glucose unit), 3.96 (dd, *J* = 10.6 and 8.6 Hz, H-6b′′′ glucose unit), 3.88 (d, *J* = 7.2 Hz, H-2′′′ glucose unit); ^13^C-NMR (100 MHz, DMSO-*d*_6_): 165.3 (C=O galloyl unit), 145.9 (C-3 and C-5 galloyl unit), 139.8 (C-4 galloyl unit), 119.3 (C-1 galloyl unit), 109.4 (C-2 and C-6 galloyl unit), 167.4 and 167.0 (C=O HHDP), 145.1 and 145.0 (C-5′ and C-5′′), 144.6 and 144.3 (C-3′ and C-3′′), 135.8 and 135.7 (C-4′ and C-4′′), 124.1 and 123.4 (C-1′ and C-1′′), 116.1 and 115.8 (C-6′ and C-6′′), 107.2 and 106.3 (C-2′ and C-2′′), 92.5 (C-1′′′ anomeric carbon), 77.8 (C-3′′′), 76.6 (C-5′′′), 71.9 (C-2′′′), 62.4 (C-4′′′), 64.2 (C-6′′′).

### 3.5. Determination of Total Polyphenolic Compounds and Total Contents of Flavonoids 

Each dried root extract (1 g each) of *G. collinum* was reconstituted in 20 mL of 70% methanol and this extract was used for the determination of total polyphenolic compounds and total flavonoids contents. Total polyphenolic compounds were determined by Folin-Ciocalteau [25,26] method using gallic acid as the reference standard in the concentration range of 0.02 mg/mL to 0.2 mg/mL in water. Amount of total flavonoids were estimated using the procedure adopted by Numonov et al. [27]. 

### 3.6. Antidiaibetic Activity: PTP-1B Enzymatic Assay 

The enzyme activity was measured using the PTP-1B (3-(3,5-dibromo-4-hydroxybenzoyl)-2-ethylbenzofuran-6-sulfonic acid-(4-(thiazol-2-ylsulfamyl)-phenyl)-amide) enzyme inhibition assay according to the literature [13,28,29,30].

### 3.7. Molecular Docking

Protein-ligand docking studies were carried out based on the structures of two human protein tyrosine phosphatase 1B crystal structures (HsPTP1B, PDB 3CWE [31] and PDB 4Y14) [32] and two α-glucosidase crystal structures (human sucrose-isomaltase, HsSI, PDB 3LPP [33], and human maltase-glucoamylase, HsMGAM, PDB 3TOP [34]). Prior to docking, all solvent molecules and the co-crystallized ligands were removed from the structures. Molecular docking calculations for all compounds with each of the proteins were undertaken using Molegro Virtual Docker (version 6.0.1, Molegro ApS, Aarhus, Denmark) [35], with a sphere (15 Å radius) large enough to accommodate the cavity centered on the binding sites of each protein structure in order to allow each ligand to search. Standard protonation states of the proteins based on neutral pH were used in the docking studies. Each protein was used as a rigid model structure; no relaxation of the protein was performed. Assignments of the charges on each protein were based on standard templates as part of the Molegro Virtual Docker program; no other charges were necessary to be set. Each ligand structure was built using Spartan16 for Windows (version 2.0.1, Wavefunction Inc., Irvine, CA, USA). For each ligand, a conformational search and geometry optimization was carried out using the MMFF force field [36]. Flexible ligand models were used in the docking and subsequent optimization scheme. Variable orientations of each of the ligands were searched and ranked based on their re-rank score. For each docking simulation the maximum number of iterations for the docking algorithm was set to 1500, with a maximum population size of 50, and 30 runs per ligand. The RMSD threshold for multiple poses was set to 1.00 Å. The generated poses from each ligand were sorted by the calculated re-rank score. In order to correct for the known biasing of docking energies (Edock) with increasing molecular weight (MW) [37], we have also determined a normalized docking score (DS_norm_) based on the molecular weight: DS_norm_ = 7.2 × Edock/MW⅓ [38].

### 3.8. α-Glucosidase Inhibition Assay

α-Glucosidase inhibitory activities were evaluated according to the chromogenic method described by [39].

### 3.9. Antioxidant Activity

Antioxidant activity was measured using the DPPH (1,1-diphenyl-2-picrylhydrazyl) scavenging assay procedure as published in the literature [40,41]. The extracts were dissolved in DMSO in a concentration of 100 µg/mL. Different concentrations of each extract were prepared in DMSO. Sample solution in different concentrations was added at 2.5 mL in each well of the 96 micro well plate. Into each well was added 1 mL of 0.3 mM DPPH solution in ethanol to produce the test solutions. DMSO (1 mL) was added to produce the blank solutions. Negative control sample was prepared by mixing 1 mL of DPPH solution with 2.5 mL of DMSO. The solutions were kept in the dark at room temperature for 30 min. Absorbance was measured at the wavelength of 517 nm. Ascorbic acid (vitamin C) was used as the standard sample. 

## 4. Conclusions

The underground part of *G. collinum* growing in Tajikistan can provide a natural source of antidibetical drugs and antioxidants. Our investigations suggest the development of herbal formulations based on the roots parts of *G. collinum* growing in Tajikistan, however, further toxicological and dose determination studies are required to realize an effective phytopharmaceutical. The 50% ethanol extract delivered 10 pure biologically active compounds. Based on molecular docking, we conclude that polyphenolic constituents are likely responsible for the inhibition of PTP-1B while the sterol glucoside (daucosterol) is a likely inhibitor of human α-glucosidase. Our investigation clearly showed that other fractions also have potent activities and deserve further investigation in the future. In the present investigation, phenolic compounds and steroid glycoside were identified for the first time from roots of *G. collinum*. The alcoholic extracts of *G. collinum* exhibited strong antidiabetic activity.

## Figures and Tables

**Table 1:** Results of the total polyphenolic compounds, total flavonoids, antioxidant and antidiabetic activities.

Sample	Total Polyphenolic Compounds, mg GAE/g Extract	Total Flavonoids, mg QE/g Extract	Antioxidant Activity IC_50_ Values (μg/mL)	Antidiabetic Activity (PTP-1B) IC_50_ Values (μg/mL)	Antidiabetic Activity (α-Glucosidase) IC_50_ Values (μg/mL)
H_2_O	12.21 ± 0.10	3.31 ± 0.04	15.17 ± 0.84	0.13 ± 0.01	0.11 ± 0.01
30% EtOH	83.74 ± 0.18	42.77 ± 0.12	10.89 ± 0.63	0.29 ± 0.02	0.10 ± 0.01
50% EtOH	349.84 ± 0.21	96.07 ± 0.08	11.21 ± 0.49	0.10 ± 0.01	0.07 ± 0.01
70% EtOH	180.14 ± 0.11	75.31 ± 0.07	12.69 ± 0.6	0.16 ± 0.01	0.09 ± 0.01
EtOH absolute	100.42 ± 0.14	55.68 ± 0.02	11.23 ± 0.7	0.43 ± 0.02	1.98 ± 0.21
Vitamin C			5.34 ± 0.42		
PTP-1B ^a^ inhibitor				1.46 ± 0.40	
α-Glucosidase ^b^					2.19 ± 0.04

Note: ^a^—PTP-1B-enzyme inhibitor (3-(3,5-dibromo-4-hydroxybenzoyl)-2-ethylbenzofuran-6-sulfonic acid-(4-(thiazol-2-ylsulfamyl)-phenyl)-amide); ^b^—α-glucosidase enzyme inhibitor (acarbose).

**Table 2:** In vitro antidiabetic activity (as IC_50_ values) of isolated major compounds from *Geranium collinum.*

Name of Compounds	Antidiabetic Activity	
	(PTP-1B) IC_50_ Values (μg/mL)	(α-Glucosidase) IC_50_ Values (μg/mL)
3,3′,4,4′-Tetra-*O*-methylellagic acid	21.64 ± 1.19	No effect
3,3′-Di-*O*-methylellagic acid	6.26 ± 0.22	No effect
Caffeic acid	35.81 ± 1.62	22.49 ± 1.12
Quercetin	2.19 ± 0.2	4.15 ± 0.19
Catechin	0.62 ± 0.06	4.65 ± 0.20
Epicatechin	0.23 ± 0.04	2.62 ± 0.12
Epigallocatechin	No effect	42.44 ± 2.15
Gallic acid	No effect	68.30 ± 3.02
Daucosterol	No effect	30.19 ± 1.56
Corilagin	0.87 ± 0.09	5.59 ± 0.37
PTP-1B ^a^ and acarbose ^b^	1.46 ± 0.40	2.19 ± 0.11

Note: ^a^—PTP-1B-enzyme inhibitor (3-(3,5-dibromo-4-hydroxybenzoyl)-2-ethylbenzofuran-6-sulfonic acid-(4-(thiazol-2-ylsulfamyl)-phenyl)-amide); ^b^—α-glucosidase enzyme inhibitor (acarbose).

**Table 3:** Molegro docking energies (re-rank scores, E_dock_) and normalized docking scores (DS_norm_), kJ/mol, for *Geranium collinum* phytochemicals with human protein tyrosine phosphatase 1B and human α-glucosidase.

Ligand	Protein Tyrosine Phosphatase 1B				α-Glucosidase			
	3CWE ^a^		4Y14 ^b^		3LPP ^c^		3TOP ^d^	
	E_dock_	DS_norm_	E_dock_	DS_norm_	E_dock_	DS_norm_	E_dock_	DS_norm_
Co-crystallized ligand	−87.4	−79.5	−125.0	−116.1	−116.0	−110.9	−130.3	−108.3
3,3′,4,4′-Tetra-*O*-methylellagic acid	−91.4	−92.5	−98.6	−99.8	−55.9	−56.6	−83.2	−84.2
3,3′-Di-*O*-methylellagic acid	−82.4	−85.7	−93.3	−97.0	−59.8	−62.2	−72.2	−75.1
Caffeic acid	−78.2	−99.6	−81.5	−103.8	−69.6	−88.6	−65.9	−83.9
Catechin	−86.9	−94.3	−86.9	−94.4	−77.5	−84.1	−83.5	−90.7
Corilagin	−111.9	−93.7	−101.2	−84.4	−107.5	−89.9	−120.4	−100.8
Daucosterol	−12.6	−10.8	−86.4	−74.6	−103.4	−89.3	−129.3	−111.6
Ellagic acid	−82.3	−88.1	−92.3	−98.9	−51.7	−55.4	−72.5	−77.6
Epicatechin	−87.4	−94.9	−95.7	−103.9	−76.5	−83.1	−82.3	−89.4
Epigallocatechin	−91.9	−98.0	−98.5	−105.1	−79.8	−85.1	−89.2	−95.1
Gallic acid	−75.5	−97.9	−78.8	−102.3	−70.4	−91.3	−67.0	−86.9
Quercetin	−93.4	−100.0	−92.6	−99.2	−84.7	−90.7	−87.7	−93.9

^a^ The co-crystallized ligand for PDB 3CWE is [{2-bromo-4-[(2*R*)-3-oxo-2,3-diphenylpropyl]phenyl}(difluoro)-methyl]phosphonic acid (MW = 494.243); ^b^ the co-crystallized ligand for PDB 4Y14 is 3-bromo-4-[difluoro-(phosphono)methyl]-*N*-methyl-*N*α-(methylsulfonyl)-l-phenylalaninamide (MW = 463.189); ^c^ the co-crystallized ligand for PDB 3LPP is (1*S*,2*R*,3*R*,4*S*)-1-{(1*S*)-2-[(2*R*,3*S*,4*S*)-3,4-dihydroxy-2-(hydroxymethyl)tetrahydrothio-phenium-1-yl]-1-hydroxyethyl}-2,3,4,5-tetrahydroxypentyl sulfate (MW = 424.442); ^d^ the co-crystallized ligand for PDB 3TOP is α-acarbose (MW = 646.613).

## References

[1] Bhandari M.R., Jong-Anurakkun N., Hong G., Kawabata J. (2008). α-Glucosidase and α-amylase inhibitory activities of Nepalese medicinal herb Pakhanbhed (Bergenia ciliata, Haw.). Food Chem..

[2] Asase A., Kokubun T., Grayer R.J., Kite G., Simmonds M.S.J., Oteng-Yeboah A.A., Odamtten G.T. (2008). Chemical constituents and antimicrobial activity of medicinal plants from Ghana: *Cassia sieberiana*, *Haematostaphis barteri*, *Mitragyna inermis* and *Pseudocedrela kotschyi*. Phytother. Res..

[3] De Sousa E., Zanatta L., Seifriz I., Creczynski-Pasa T.B., Pizzolatti M.G., Szpoganicz B., Silva F.R.M.B. (2004). Hypoglycemic effect and antioxidant potential of kaempferol-3,7-*O*-(α)-dirhamnoside from *Bauhinia forficata* leaves. J. Nat. Prod..

[4] Jung M., Park M., Lee H.C., Kang Y.-H., Kang E.S., Kim S.K. (2006). Antidiabetic agents from medicinal plants. Curr. Med. Chem..

[5] Tang D., Chen Q.-B., Xin X.-L., Aisa H.-A. (2017). Anti-diabetic effect of three new norditerpenoid alkaloids in vitro and potential mechanism via PI3K/Akt signaling pathway. Biomed. Pharmacother..

[6] Chakroun M., Khemakhem B., Mabrouk H.B., El Abed H., Makni M., Bouaziz M., Drira N., Marrakchi N., Mejdoub H. (2016). Evaluation of anti-diabetic and anti-tumoral activities of bioactive compounds from *Phoenix dactylifera* L’s leaf: In vitro and in vivo approach. Biomed. Pharmacother..

[7] Shibkova I.F., Klyzikaeva K.G. (1981). Geran Anchabir (tadj) Geranium Z, Flora of Tajik SSR. Science.

[8] Hodzhimatov M. (1989). Wild Medicinal Plants of Tajikistan. Tajik Soviet Encyclopedia.

[9] Saleh N.A.M., El-karemy Z.A.R., Mansour R.M.A., Fayed A.-A.A. (1983). A chemosystematic study of some geraniaceae. Phytochemistry.

[10] Elhassan G.O.M., Adhikari A., Abdalla O.M., Shukrulla A., Khalid A., Iqbal Choudhary M., Mesaik M.A., Yagi S. (2015). Chemical constituents of euphorbia polyacantha Boiss. and their immunomodulatory properties. Rec. Nat. Prod..

[11] Sata T. (1987). Spectral differentiation of 3,3′-di-*O*-methylellagic acid from 4,4′-di-O-methylellagic acid. Phytochemistry.

[12] Chang S.W., Kim K.H., Lee I.K., Choi S.U., Ryu S.Y., Lee K.R. (2009). Phytochemical constituents of *Bistorta manshuriensis*. Nat. Prod. Sci..

[13] Jiang L., Numonov S., Bobakulov K., Qureshi M., Zhao H., Aisa H. (2015). Phytochemical profiling and evaluation of pharmacological activities of *Hypericum scabrum* L.. Molecules.

[14] Qureshi M.N., Numonov S., Abudurexiti A., Aisa H.A. (2016). Phytochemical investigations and evaluation of antidiabetic potential of *Prunus dulcis* nuts. LWT Food Sci. Technol..

[15] Zang X., Shang M., Xu F., Liang J., Wang X., Mikage M., Cai S. (2013). A-Type proanthocyanidins from the stems of *Ephedra sinica* (Ephedraceae) and their antimicrobial activities. Molecules.

[16] Liu M., Yang S., Jin L., Hu D., Wu Z., Yang S. (2012). Chemical constituents of the ethyl acetate extract of *Belamcanda chinensis* (L.) DC roots and their antitumor activities. Molecules.

[17] Numonov S.R., Usmanova S.K., Aisa H.A. (2013). Chemical composition of *Dracocephalum heterophyllum*. Chem. Nat. Compd..

[18] Conegero L.D.S., Ide R.M., Nazari A.S., Sarragiotto M.H., Dias Filho B.P., Nakamura C.V., Carvalho J.E.D., Foglio M.A. (2003). Constituintes químicos de *Alchornea glandulosa* (Euphorbiaceae). Quim. Nova.

[19] Yen G.-C., Chen C.-S., Chang W.-T., Wu M.-F., Cheng F.-T., Shiau D.-K., Hsu C.-L. (2017). Antioxidant activity and anticancer effect of ethanolic and aqueous extracts of the roots of *Ficus beecheyana* and their phenolic components. J. Food Drug Anal..

[20] Li Q., Zhang X., Cao J., Guo Z., Lou Y., Ding M., Zhao Y. (2015). Depside derivatives with anti-hepatic fibrosis and anti-diabetic activities from *Impatiens balsamina* L. flowers. Fitoterapia.

[21] Zhang J., Shen Q., Lu J.-C., Li J.-Y., Liu W.-Y., Yang J.-J., Li J., Xiao K. (2010). Phenolic compounds from the leaves of *Cyclocarya paliurus* (Batal.) Ijinskaja and their inhibitory activity against PTP1B. Food Chem..

[22] Oboh G., Ogunsuyi O.B., Ogunbadejo M.D., Adefegha S.A. (2016). Influence of gallic acid on α-amylase and α-glucosidase inhibitory properties of acarbose. J. Food Drug Anal..

[23] Tiong S., Looi C., Hazni H., Arya A., Paydar M., Wong W., Cheah S.-C., Mustafa M., Awang K. (2013). Antidiabetic and antioxidant properties of alkaloids from *Catharanthus roseus* (L.) G. Don. Molecules.

[24] Umeno A., Horie M., Murotomi K., Nakajima Y., Yoshida Y. (2016). Antioxidative and antidiabetic effects of natural polyphenols and isoflavones. Molecules.

[25] Phosrithong N., Nuchtavorn N. (2016). Antioxidant and anti-inflammatory activites of *Clerodendrum* leaf extracts collected in Thailand. Eur. J. Integr. Med..

[26] Qureshi M.N., Stecher G., Bonn G.K. (2014). Determination of total polyphenolic compounds and flavonoids in *Juglans regia* leaves. Pak. J. Pharmaceut.Sci..

[27] Numonov S.R., Qureshi M.N., Aisa H.A. (2015). Development of HPLC protocol and simultaneous quantification of four free flavonoids from *Dracocephalum heterophyllum* Benth. Int. J. Anal. Chem..

[28] Ali J., Khan A., Ullah N. (2012). Essential oil chemical composition and antimicrobial activity of sour oranges (*Citrus aurantium*) peels. J. Pharm. Res..

[29] Bozorov K., Ma H.-R., Zhao J.-Y., Zhao H.-Q., Chen H., Bobakulov K., Xin X.-L., Elmuradov B., Shakhidoyatov K., Aisa H.A. (2014). Discovery of diethyl 2,5-diaminothiophene-3,4-dicarboxylate derivatives as potent anticancer and antimicrobial agents and screening of anti-diabetic activity: Synthesis and in vitro biological evaluation. Part 1. Eur. J. Med. Chem..

[30] Yili A., Yimamu H., Ghulameden S., Qing Z., Aisa H., Morlock G. (2014). Determination of antidiabetic polysaccharides of *Ocimum basilicum* seeds indigenous to xinjiang of China by high-performance thin-layer chromatography-UV/Vis-mass spectrometry. J. Planar Chromat. Modern TLC.

[31] Han Y., Belley M., Bayly C.I., Colucci J., Dufresne C., Giroux A., Lau C.K., Leblanc Y., McKay D., Therien M. (2008). Discovery of [(3-bromo-7-cyano-2-naphthyl)(difluoro)methyl]phosphonic acid, a potent and orally active small molecule PTP1B inhibitor. Bioorg. Med. Chem. Lett..

[32] Krishnan N., Krishnan K., Connors C.R., Choy M.S., Page R., Peti W., Van Aelst L., Shea S.D., Tonks N.K. (2015). PTP1B inhibition suggests a therapeutic strategy for Rett syndrome. J. Clin. Investig..

[33] Sim L., Willemsma C., Mohan S., Naim H.Y., Pinto B.M., Rose D.R. (2010). Structural basis for substrate selectivity in human maltase-glucoamylase and sucrase-isomaltase N-terminal domains. J. Biol. Chem..

[34] Ren L., Qin X., Cao X., Wang L., Bai F., Bai G., Shen Y. (2011). Structural insight into substrate specificity of human intestinal maltase-glucoamylase. Protein Cell.

[35] Thomsen R., Christensen M.H. (2006). MolDock: A new technique for high-accuracy molecular docking. J. Med. Chem..

[36] Halgren T.A. (1996). Merck molecular force field. I. Basis, form, scope, parameterization, and performance of MMFF94. J. Comput. Chem..

[37] Pan Y., Huang N., Cho S., MacKerell A.D. (2003). Consideration of molecular weight during compound selection in virtual target-based database screening. J. Chem. Inf. Comput. Sci..

[38] Snow Setzer M., Sharifi-Rad J., Setzer W. (2016). The search for herbal antibiotics: An in-silico investigation of antibacterial phytochemicals. Antibiotics.

[39] McCue P., Kwon Y.I., Shetty K. (2005). Anti-diabetic and anti-hypertensive potential of sprouted and solid-state bioprocessed soybean. Asia Pac. J. Clin. Nutr..

[40] Jaradat N.A., Shawahna R., Hussein F., Al-Lahham S. (2016). Analysis of the antioxidant potential in aerial parts of *Trigonella arabica* and *Trigonella berythea* grown widely in Palestine: A comparative study. Eur. J. Integr. Med..

[41] Zhao Y., Dou J., Wu T., Aisa H. (2013). Investigating the antioxidant and acetylcholinesterase inhibition activities of *Gossypium herbaceam*. Molecules.

